# Virulence-related gene *wx2* of *Toxoplasma gondii* regulated host immune response via classic pyroptosis pathway

**DOI:** 10.1186/s13071-022-05502-5

**Published:** 2022-12-05

**Authors:** Zhenrong Ma, Zhuolin Li, Ruolan Jiang, Xuanwu Li, Kang Yan, Ni Zhang, Bin Lu, Yehong Huang, Nouhoum Dibo, Xiang Wu

**Affiliations:** 1 Department of Parasitology, Xiangya School of Basic Medicine, Central South University, Changsha, China; 2 School of Public Health, Southern Medical University, Guangzhou, China; 3 Hunan Provincial Key Lab of Immunology and Transmission Control On Schistosomiasis, Changsha, China

**Keywords:** *wx2* gene, *Toxoplasma gondii*, Virulence, Pyroptosis, Host immune response

## Abstract

**Background:**

*Toxoplasma gondii* is known as the most successful parasite, which can regulate the host immune response through a variety of ways to achieve immune escape. We previously reported that a novel gene *wx2* of *T. gondii* may be a virulence-related molecule. The objective of this study was to explore the mechanism of *wx2* regulating host immune response.

**Methods:**

The *wx2* knockout strain (RH^*wx2−/−*^ strain) and complementary strain (RH^*wx2*+*/*+^ strain) were constructed by the CRISPR/Cas9 technique, and the virulence of the *wx2* gene was detected and changes in pyroptosis-related molecules were observed.

**Results:**

Compared with the wild RH and RH^*wx2*+*/*+^ strain groups, the survival time for mice infected with the RH^*wx2−/−*^ strain was prolonged to a certain extent. The mRNA levels of pyroptosis-related molecules of caspase-1, NLRP3, and GSDMD and et al. in mouse lymphocytes in vivo and RAW267.4 cells in vitro infected with RH^*wx2−/−*^ strain increased to different degrees, compared with infected with wild RH strain and RH^*wx2*+*/*+^ strain. As with the mRNA level, the protein level of caspase-1, caspase-1 p20, IL-1β, NLRP3, GSDMD-FL, GSDMD-N, and phosphorylation level of NF-κB (p65) were also significantly increased. These data suggest that *wx2* may regulate the host immune response through the pyroptosis pathway. In infected RAW264.7 cells at 48 h post-infection, the levels of Th1-type cytokines of IFN-γ, Th2-type cytokines such as IL-13, Th17-type cytokine of IL-17 in cells infected with RH^*wx2−/−*^ were significantly higher than those of RH and RH^*wx2*+*/*+^ strains, suggesting that the *wx2* may inhibit the host's immune response.

**Conclusion:**

*wx2* is a virulence related gene of *T. gondii,* and may be involved in host immune regulation by inhibiting the pyroptosis pathway.

**Graphical Abstract:**

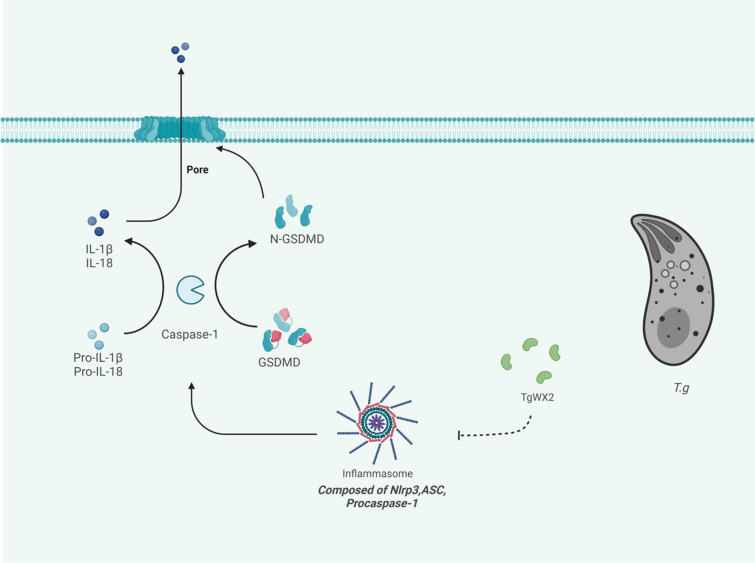

**Supplementary Information:**

The online version contains supplementary material available at 10.1186/s13071-022-05502-5.

## Background

*Toxoplasma gondii* is an obligate intracellular parasite of Apicomplexa, which can infect almost all warm-blooded animals, including humans [1, 2]. *Toxoplasma gondii* is widely distributed throughout the world, with about one third of the world’s population infected [3]. Although *T. gondii* has been known for more than 100 years, the mechanism of its invasion is not fully understood. How the host controls the parasite and the mechanisms by which the parasite resists the host’s immune response are unclear. To date, there are no effective drugs against *T. gondii* bradyzoites. In addition, drug-resistant *T. gondii* strains have been reported by researchers [4]. Therefore, research is still needed for an in-depth understanding of the pathogenic mechanism of *T. gondii* to develop effective drugs and vaccines.

The host has a variety of immune mechanisms to resist *T. gondii* infection. The invading *T. gondii* are mainly eliminated via helper T type 1 (Th1) cellular immunity [5]. In the early stage of infection, the *T. gondii* antigen can induce interleukin-12 (IL-12) secretion in dendritic cells [6, 7]. In addition, neutrophils and macrophages can also secrete IL-12 during *T. gondii* infection [8, 9]. IL-12 plays an important role in *T. gondii* infection. It can stimulate natural killer (NK) cells, CD4^+^, and CD8^+^ T cells to secrete interferon-γ (IFN-γ), which is the most important cytokine for the host to fight against *T. gondii* [5, 6, 10].

During the long-term parasitic process, to avoid being eradicated by the host immune response, *T. gondii* has also evolved a series of immune evasion mechanisms to protect itself from being eliminated [11]. After *T. gondii* invades the cells, it is located in the cytoplasm of the host cells and is wrapped by a parasitophorous vacuole membrane (PVM). The PVM can act as a molecular sieve [12], so that *T. gondii* can secrete the parasite's specialized secretory proteins, such as the rhoptries (ROP) or dense granules (GRA) [13, 14], which play an important role in the modulation of host signaling pathways to achieve the purpose of immune escape. Activators of transcription (STAT) such as STAT3 and STAT6 of host cells were activated by ROP16 [15, 16], which inhibits lipopolysaccharide (LPS)-induced production of IL-12p40 and tumor necrosis factor (TNF-α) [17]. ROP16 is also a major inhibitor of Toll-like receptors (TLR) and IFN-γ downstream pathways [18]. The inhibitor of STAT1-dependent transcription (TgIST [*Toxoplasma* inhibitor of STAT1-dependent transcription]) is a dense granule protein, which can also inhibit the expression of STAT1-dependent proinflammatory genes, such as IFN-γ [19]. In addition, *T. gondii* can also impact the host NF-κB, MAPK and other pathways through molecules such as GRA15 and GRA24 [20] to modulate host signaling pathways. *Toxoplasma gondii* can also evade host immune response by inhibiting host cell apoptosis, affecting the host cell cycle and cell metabolism [21, 22].

Pyroptosis is a pro-inflammatory form of programmed cell death that is regulated by the cysteine-dependent caspase family of proteases. Different from cell necrosis, apoptosis, and autophagy, it is manifested in the continuous swelling of cells until the cell membrane ruptures, resulting in the release of cell contents and the activation of a strong inflammatory response. Pyroptosis was first discovered in the lysis reaction after *Shigella* infection of macrophages in 1992 [23], and in 2001, Cookson et al. [24] named it pyroptosis. Studies have confirmed that both pathogenic infection and endogenous damage can induce pyroptosis.

The pathways of pyroptosis can be divided into two categories: the caspase-1-dependent classical pathway and the caspase-1-independent non-canonical pathway, both of which perforate the cell membrane through the regulation of gasdermin D (GSDMD). Inactive caspase-1 exists in the cytoplasm in the form of pro-caspase-1, and its activation depends on the inflammasome action. Nod-like receptor (NLR) is the earliest discovered inflammasome family, including multiple members such as NLRP1, NLRP3, NAIP-NLRC4, NLRP6, and NLRP9b [25]. And NLRP3 is not only the main component of the classical pyroptosis pathway but also an important component of the non-classical pyroptosis pathway [26]. When the host is subjected to exogenous or endogenous damage, stimulated by pathogen-associated molecular patterns/damage-associated molecular patterns/hepcidin antimicrobial peptide (PAMP/DAMP/ HAMP), NLRP3 is activated, recruits apoptosis-associated speck-like protein (ASC) and pro-caspase-1, and assembles into a large cytoplasmic complex, pro-caspase-1 release p20 and p10 subunits undergo autohydrolysis, and further aggregates into active caspase-1. Activated caspase-1 cleaves GSDMD into N-terminal and C-terminal, leading to the formation of active pores in the cell membrane, and further cell membrane perforation occurs until cell lysis and death [27]. At the same time, caspase-1 cleaves the precursors of IL-1β and IL-18 to form mature IL-1β and IL-18; after the cell membrane is perforated, IL-1β and IL-18 are secreted out of the cell via porin, inducing an inflammatory response, and activating the pyroptosis of other cells [28].

Pyroptosis is also one of the ways that the host eliminates various intracellular pathogens, including *T. gondii*. However, *T. gondii* has also developed some strategies to inhibit host cell pyroptosis. For example, the C-terminal of GRA9 secreted by *T. gondii* can bind to NLRP3 and thus block the binding of ASC to NLRP3, thereby destroying the NLRP3 inflammasome [29]. Studies have found that *T. gondii* could inhibit the activity of caspase-1 in neutrophils and may also affect the activity of GSDMD [30, 31].

Our previous studies have shown that the *wx2* gene may be related to *T. gondii* virulence and may even regulate the host immune response to achieve immune escaping [32]. In this study, we explore whether the *wx2* gene of *T. gondii* affects the host immune response via the pyroptosis pathway in vitro and in vivo. It provides insights into the mechanism of host immune response manipulation by *T. gondii* that might be useful in the development of anti-toxoplasmosis drugs and vaccines.

## Methods

### *Toxoplasma gondii* and cell culture

Tachyzoites of *T. gondii* RH strain (type I), *wx2* knockout (RH^*wx2−/−*^ strain), and *wx2* complementary (RH^*wx2*+*/*+^ strain) were maintained in Kun Ming (KM) mice and were harvested in phosphate-buffered saline (PBS) solution after infection for 4 days.

Tachyzoites of *T. gondii* RH strain (type I), *wx2* knockout (RH^*wx2−/−*^ strain), and *wx2* complementary (RH^*wx2*+*/*+^ strain) were cultured in human foreskin fibroblasts (HFF) using Dulbecco’s modified Eagle medium (DMEM) supplemented with 2% fetal bovine serum (FBS). HFF cells were grown in culture flasks containing DMEM supplemented with 10% FBS, in a 37 °C and 5% CO_2_ incubator.

### Construction of *T. gondii wx2* gene complementary strains

Tachyzoites of *T. gondii* RH strain (type I) and *wx2* knockout (RH^*wx2−/−*^ strain) were stored in our laboratory. The *wx2* gene complementary strains were constructed as described in the literature using CRISPR-Cas9 [32, 33]. Ptub::GOI::CAT plasmid with the *wx2* fragment and purified *wx2*-CAT fragment were co-transfected into freshly harvested tachyzoites of RH^*wx2−/−*^ strain by electroporation. The transgenic parasites were obtained by selection via 25 µg/ml pyrimethamine chloramphenicol (CAT) and 10 µg/ml fluorouracil deoxynucleoside (FUDR). Polymerase chain reaction (PCR) was performed with genomic DNA as a template to confirm the gene of *wx2* was complemented. All plasmids and primers used in this study are listed in Table 1.

**Table 1:** Information of primers

Name of primer	Forward (5′-3′)	Reverse (5′-3′)
*wx2*-CDS	ATGTATATCTGTATAGAAGG	CGGTGTCGCCTGACTTCTGT
pTub-Gbison	TACCCGTACGACGTCCCGGACTACGCTGGCTATCCC	TTTGTCGAAAAAGGGAATTCAAGAAAA
Com-UPRT-*wx2*-KZ	TCCTTTTATTCCAAGATCTGTGGCGTCTCGATTGTGAGGAAGTGGAGGACGGGAATTCG	AAACTGCCCGCAAGCCACTTTCCATCGACTCGCCAGCTAATACGACTCACTATAGGGCG
JD1-*wx2*	TCCGTAAAGCGGTGAGTGTCG	CGGTGTCGCCTGACTTCTGTG
JD2-*wx2*	AAACATCCGTAAAGCGGTGAG	ACGACGAAGAAGGGAACACG
pro-caspase-1	CACAGCTCTGGAGATGGTGA	CTTTCAAGCTTGGGCACTTC
ASC	GACAGTACCAGGCAGTTCGT	AGTCCTTGCAGGTCAGGTTC
pro-IL-1β	CAGGCAGGCAGTATCACTCA	AGCTCATATGGGTCCGACAG
NF-κB	GAGGAAGGCTGTGAACATGAGG	TTCTGGTGCATTCTGACCTTGC
NLRP3	AGATTACCCGCCCGAGAAAG	TCCCAGCAAACCCATCCACT
β-actin	TTCCTTCCTTGGGTATGGAAT	GAGCAATGATCTTGATCTTC

### Virulence assay

Specific-pathogen-free (SPF) inbred female KM mice (8 weeks old) were purchased from the Center of Laboratory of Animals, Central South University, Hunan, China. All mice were handled in strict accordance with the guidelines of the People’s Republic of China and the university’s ethics committee. Each mouse was injected intraperitoneally with 5000 freshly harvested tachyzoites of the RH, RH^*wx2−/−*^, and RH^*wx2*+*/*+^ strains (eight mice per strain). All mice were monitored daily until death.

### Detection of pyroptosis-related molecules at the mRNA level

The *wx2* gene was confirmed to be restored by reverse transcription PCR (RT-PCR) at the mRNA level. Total RNA was extracted from the RH, RH^*wx2−/−*^ strain, and RH^*wx2*+*/*+^ *T. gondii* strain using TRIzol (Vazyme Biotech Co., Ltd.) according to the manufacturer’s recommendations. Reverse transcription was performed using a PrimeScript™ 1st Strand cDNA Synthesis Kit (Vazyme Biotech Co.,Ltd.). The mRNA expression levels of *Tgwx2* was detected by quantitative RT-PCR. When the BALB/c mice were infected with the RH, RH^*wx2−/−*^, and RH^*wx2*+*/*+^ strains respectively, until death. The lymphoid tissues were taken and the total RNA was extracted. RAW264.7 cells were infected with the RH, RH^*wx2−/*−^, and RH^*wx2*+*/*+^ strains, respectively, for 24 and 48 h. Total RNA was extracted and reverse transcription was performed. The mRNA expression levels of pro-caspase-1, ASC, NLRP3, and GSDMD were detected by RT-PCR. All primers used in this study are listed in Table 1.

### Detection of pyroptosis-related molecules at the protein level

After RH, RH^*wx2−/−*^ and RH^*wx2*+*/*+^ *T. gondii* strain infected BALB/c mice, until death. The lymphoid tissue protein was harvested. RAW264.7 cells were infected with RH, RH^*wx2−/−*^ and RH^*wx2*+*/*+^ strains, respectively, the total protein was harvested 24 h and 48 h later. The protein concentration was measured using a bicinchoninic acid protein assay kit (Beyotime Biotechnology, Inc., Shanghai, China). A total of 20 μg of proteins of RH, RH^*wx2−/−*^, and RH^*wx2*+*/*+^ were boiled with sodium dodecyl sulfate polyacrylamide gel electrophoresis (SDS-PAGE) sample loading buffer and added to SDS-PAGE for electrophoresis. The proteins were blotted onto a polyvinylidene difluoride (PVDF) membrane and blocked with 5% non-fat milk for 2 h at room temperature. Subsequently, the membrane was incubated with primary antibodies (GSDMD-N, GSDMD-FL, NLRP3, pro-caspase-1, caspase-1, ASC, IL-1β, and IL-1β p17) at 4 °C overnight. The membrane was subsequently incubated for 1 h at room temperature with the secondary antibody (horseradish peroxidase [HRP]-conjugated rabbit anti-mouse immunoglobulin G [IgG] or HRP-conjugated rabbit anti-rabbit IgG). The membrane was analyzed using an enhanced chemiluminescence (ECL) western blotting system. All antibodies used in this study are listed in Table 2.

**Table 2:** Information on antibodies

Antibody	Company and catalog
NLRP3	Recombinant Anti-NLRP3 antibody: Abcam (ab263899)
IL-1β	Recombinant Anti-IL-1 beta antibody: Abcam (ab254360)
GSDMD	Recombinant Anti-GSDMD antibody: Abcam (ab219800)
GSDMD-N	GSDMDC1 Antibody (64-Y): Santa Cruz (sc-81868)
Caspase-1	Caspase-1 (E2Z1C) Rabbit mAb:CST(#24,232)
Caspase-1 p20	Caspase-1 p20 Antibody (D-4): Santa Cruz (sc-398715)
NF-κB(p65)	NF-kB p65 Antibody: SAB catalog no: 48676
p-NF-κB (p-p65)	Phospho-NF-κB p65 (Ser536) (93H1) Rabbit mAb: CST (#3033)

### Cytokine detection

RAW264.7 cells were infected with RH, RH^*wx2−/*−^ and RH^*wx2*+*/*+^ strains for 48 h. Total RNA was extracted and reverse transcription was performed. The mRNA expression levels of IFN-γ, IL-4, IL-13, and IL-17 in RAW264.7 cells were detected by qRT-PCR.

### Statistical analysis

All experiments were performed at least in triplicate. GraphPad Prism 8 was used for statistical analyses. The differences between groups were analyzed using the *t*-test and Dunnett's multiple comparisons test, and values of *P* < 0.05 were considered statistically significant. All results are presented as mean ± SD, which represents a summary of the data from at least three experiments. Statistics symbols used are as follows: ∗ *P* < 0.05, ∗ ∗ *P* < 0.01, ∗ ∗ ∗ *P* < 0.001.

## Result

### The *wx2* gene influences parasite virulence in mice

**Fig. 1:**
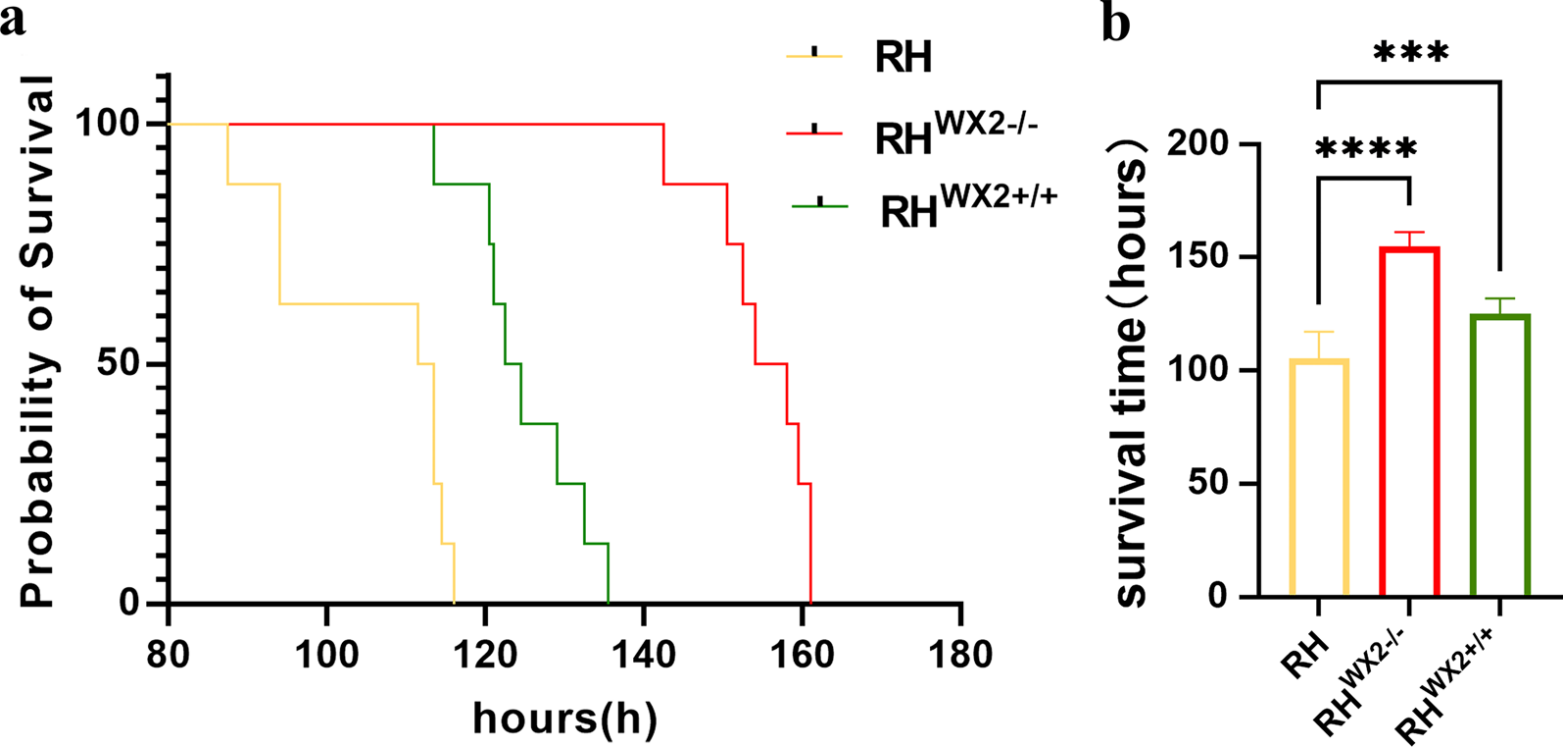
Mice virulence assay. **a** The virulence of the RH wild strain, RH^*wx2−/−*^ strain, and RH^*wx2*+*/*+^ strain was estimated using Kaplan–Meier survival analysis. **b** The histogram displays the mean survival time of RH^*wx2−/−*^, RH and RH^*wx2*+*/*+^ strain, respectively, 112.5 h, 156 h, 123.5 h

After the *wx2* gene in the complementary strain was indeed successfully complemented (Additional file 1: Fig. S1), 5000 fresh wild-type RH tachyzoites and an equal number of RH^*wx2*+*/*+^, RH^*wx2−/−*^ tachyzoites from mice were used to infect KM mice. The result showed that the average survival time for mice challenged with the RH wild strain, RH^*wx2−/−*^ strain, and RH^*wx2*+*/*+^ strain was 112.5 h, 156 h, and 123.5 h, respectively (Fig. 1a). The survival time for mice challenged with the RH^*wx2−/−*^ strain was 32.5 h longer than that of mice challenged with the RH^*wx2*+*/*+^ strain, and 43.5 h longer than that of mice challenged with the RH strain, and these differences are statistically significant (*P* < 0.05) (Fig. 1b). The above results show that the deletion of the *wx2* gene in the RH strain can delay the death of mice, and restoration of *wx2* accelerated the death of mice to a certain extent, and the difference was statistically significant(*P* < 0.05), revealing that the *wx2* gene is a virulence-related gene of *T. gondii.*

### wx2 of *T. gondii* affects host cell pyroptosis in vivo

**Fig. 2:**
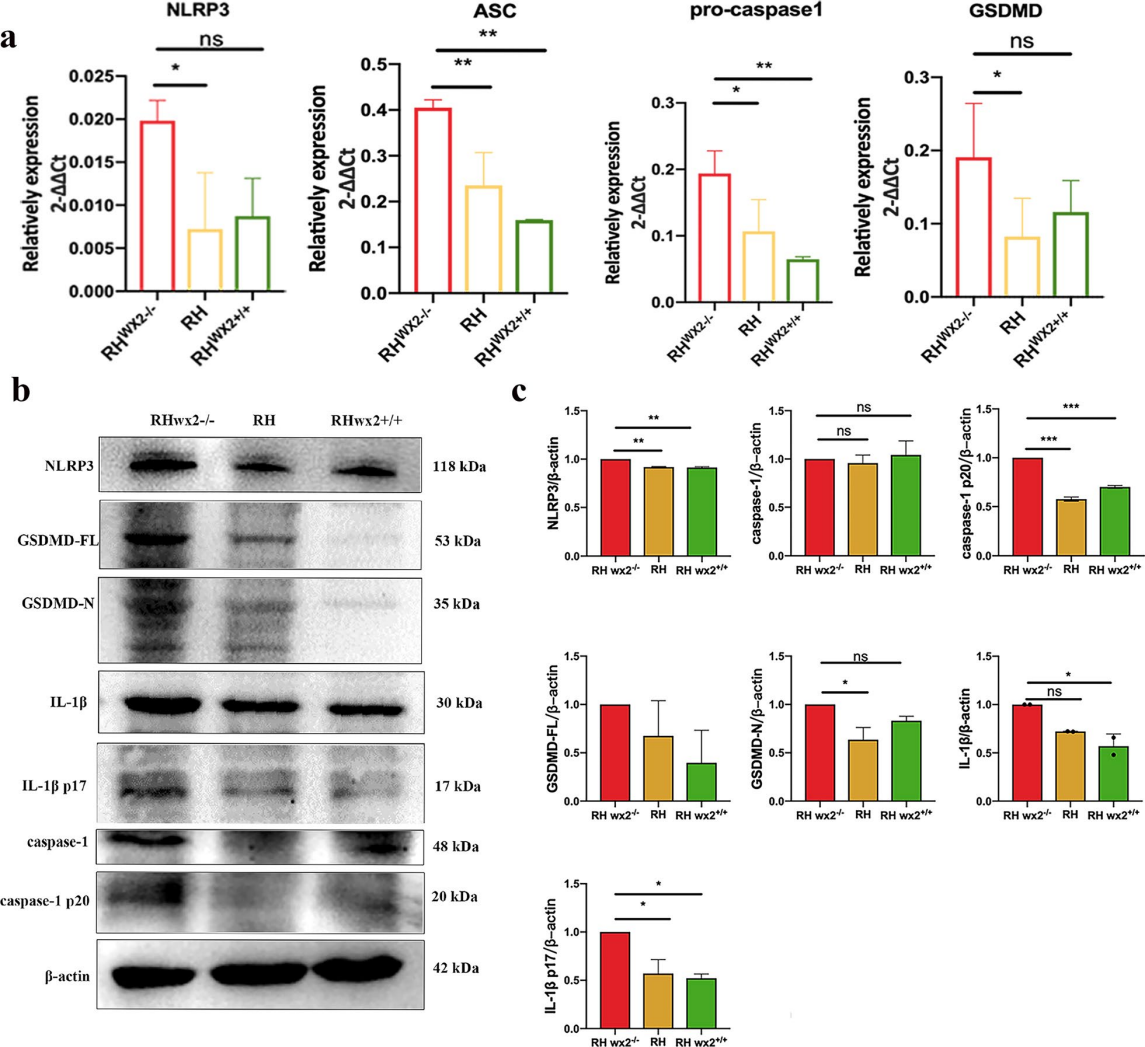
Expression of pyroptosis-related molecules in vivo. **a** Gene expression of pyroptosis-related molecules, NLRP3, ASC, pro-caspase-1, and GSDMD at mRNA levels in the lymph node of infected mice. Mice were infected with RH^*wx2−/−*^, RH, RH^*wx2*+*/*+^ strain, respectively, then total RNA was extracted, and RT-qPCR was performed with the indicated primer. **b** Protein expression of pyroptosis-related molecules of NLRP3, caspase-1, caspase-1p20, GSDMD-FL, GSDMD-N, IL-1β, IL-1β p17 in lymph node of mice infected with the RH^*wx2−/−*^, RH, and RH^*wx2*+*/*+^ strains, and western blot was performed with the indicated antibody. **c** Analysis of grayscale scanning of image B

The mRNA expression levels of NLRP3, ASC, pro-caspase-1, and gasdermin D (GSDMD) in mouse lymphocytes, as well as protein expression levels of IL-1β, IL-1β p17, caspase-1 p20, caspase-1, GSDMD-FL, and GSDMD-N were detected. Compared with the RH strain, the mRNA expression levels of NLRP3, ASC, pro-caspase-1, and GSDMD (Fig. 2a) in the lymph nodes of the RH^*wx2−/−*^ knockout mice were significantly increased, and the difference was statistically significant (*P* < 0.05), the protein expression levels of NLRP3, caspase-1 p20, GSDMD-F, GSDMD-N, IL-1β, IL-1β p17 (Fig. 2b, c) in the lymph nodes of mice infected with the *wx2* knockout strain RH^*wx2−/−*^ were also significantly higher than those of the RH and RH^*wx2*+*/*+^ strain.

### *wx2* of *T. gondii* affects host cell pyroptosis in vitro

**Fig. 3:**
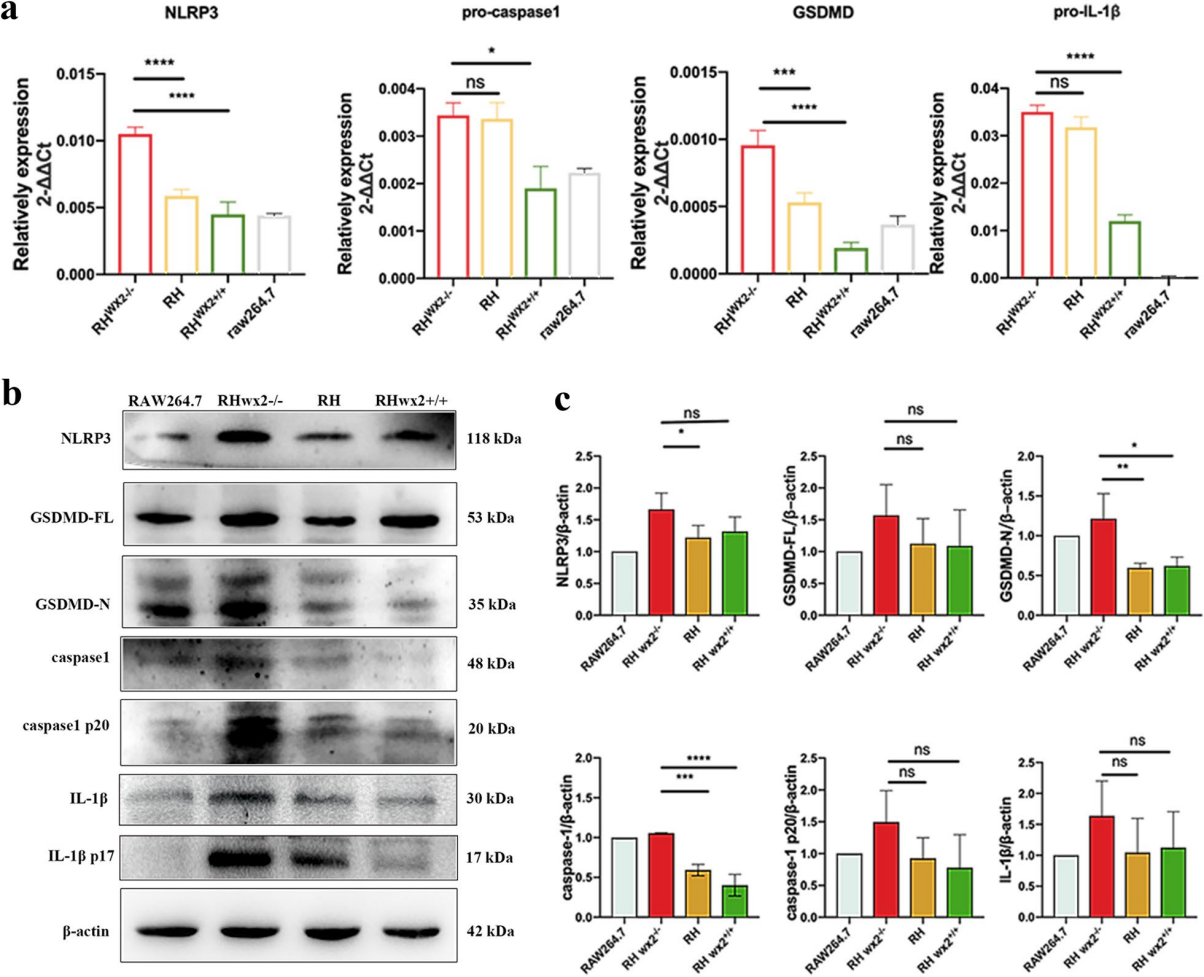
Expression of pyroptosis-related molecules in infected RAW264.7 cells at 24 h post-infection. **a** mRNA expression of pyroptosis-related molecules of NLRP3, pro-caspase-1, GSDMD, and pro-IL-1β was detected. RAW264.7 cells were infected with the RH^*wx2−/−*^, RH, and RH^*wx2*+*/*+^ strain, respectively, for 24 h, total RNA was extracted, and RT-qPCR was performed with the indicated primer. **b** Protein expression of pyroptosis-related molecules of NLRP3, pro-caspase-1, GSDMD, and pro-IL-1β was detected. **c** Analysis of grayscale scanning of image B

**Fig. 4:**
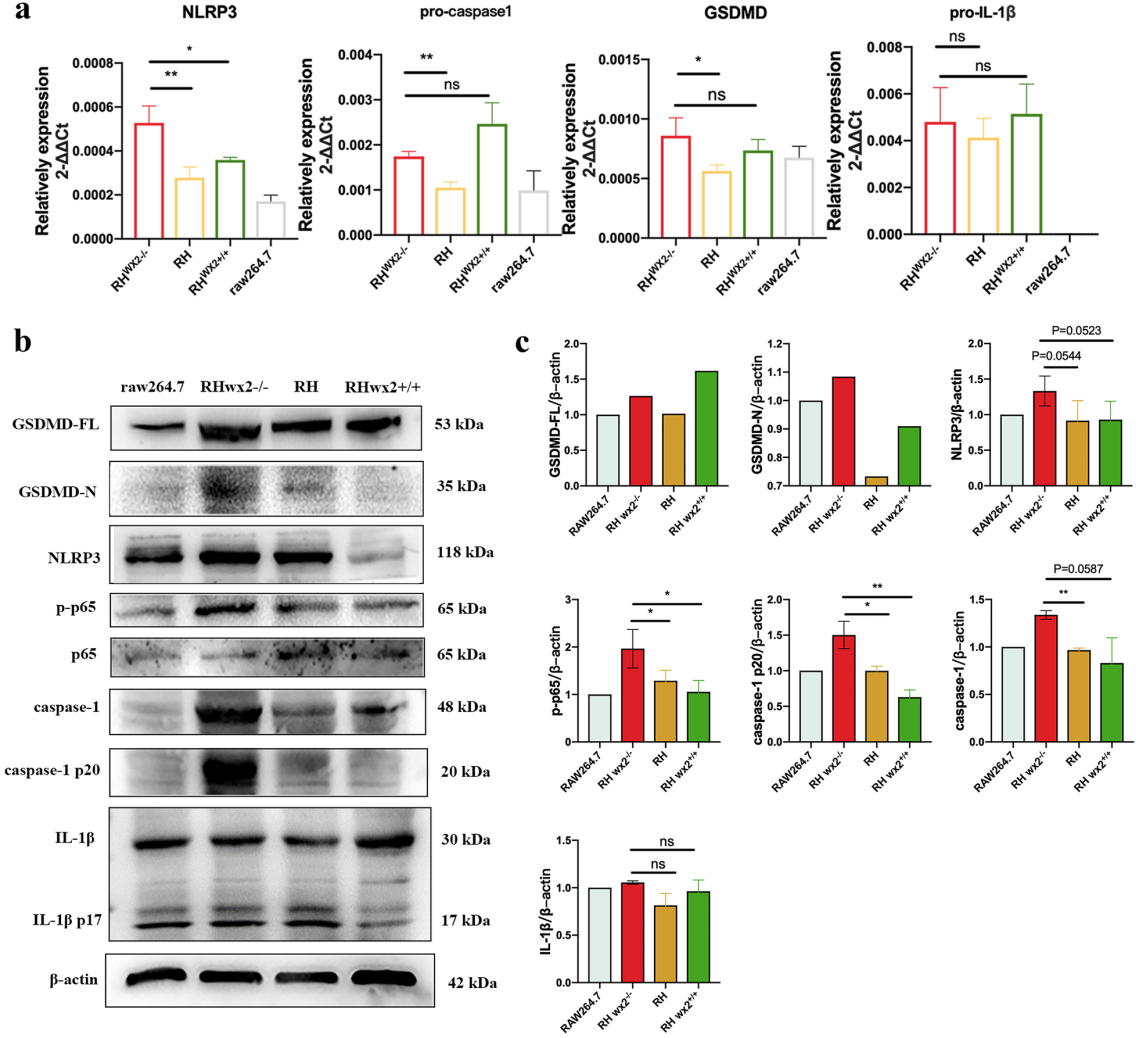
Expression of pyroptosis-related molecules in infected RAW264.7 cells at 48 h post-infection. **a** Gene expression of pyroptosis-related molecules of NLRP3, pro-IL-1β, GSDMD, and pro-caspase-1 at the mRNA level. RAW264.7 cells were infected with the RH^*wx2−/−*^, RH, and RH^*wx2*+*/*+^ strains, respectively, for 48 h, total RNA was extracted, and qRT-PCR was performed with the indicated primer. **b** Protein expression of pyroptosis-related molecules. RAW264.7 cells were infected with the RH^*wx2−/−*^, RH, and RH^*wx2*+*/*+^ strains, respectively, for 48 h, lysed, and western blot was performed with the indicated antibody, GSDMD-FL, GSDMD-N, NLRP3, p-p65, caspase-1, caspase-1 p20, and IL-1β. **c** Analysis of grayscale scanning of image B

To verify the effect of the *wx2* gene on host cell pyroptosis, we performed in vitro experiments. RAW264.7 cells were infected with RH, RH^*wx2*+*/*+^ and RH^*wx2−/*^for 24 h and 48 h, respectively, at a multiplicity of infection (MOI) of 5:1.To further verify the effect of the *wx2* gene on host cell pyroptosis, we performed in vitro experiments by infecting RAW264.7 cells with RH, RH^*wx2*+*/*+^ and RH^*wx2−/*^for 24 h and 48 h, respectively. The mRNA expression levels of pro-caspase-1, pro-IL-1β, GSDMD, and NLRP3, as well as protein levels of caspase-1, caspase-1 p20, GSDMD-N, GSDMD-FL, IL-1β in RAW264.7 cells infected with RH, RH^*wx2*+*/*+^ and RH^*wx2−/−*^ were detected after 24 h. Compared with the RH strain and RH^*wx2*+*/*+^ group, the mRNA expression levels of NLRP3, GSDMD, and pro-IL-1β (Fig. 3a)in the RAW264.7 cells infected with RH^*wx2−/−*^ were significantly increased (*P* < 0.05). As for protein expression levels, the expression levels of NLRP3, GSDMD-FL, GSDMD-N, caspase-1, caspase-1 p20, and IL-1β (Fig. 3b, c) in the RH^*wx2−/−*^ group were higher than those in the RH strain group and RH^*wx2*+*/*+^ groups. After 48 h post-infection, the mRNA expressions of NLRP3 and GSDMD (Fig. 4a) were higher in the RH^*wx2−/−*^ group compared with the RH and RH^*wx2*+*/*+^ groups, and the difference was statistically significant (*P* < 0.05). The protein expression levels of GSDMD-N, NLRP3, caspase-1, caspase-1 p20, and phosphorylation of NF-κB (p65) (Fig. 4 b, c) were also higher in the RH^*wx2−/−*^ strain than those of the infected RH strain and RH^*wx2*+*/*+^ strain.

### The *wx2* gene influences the host cell immune response in vitro

**Fig. 5:**
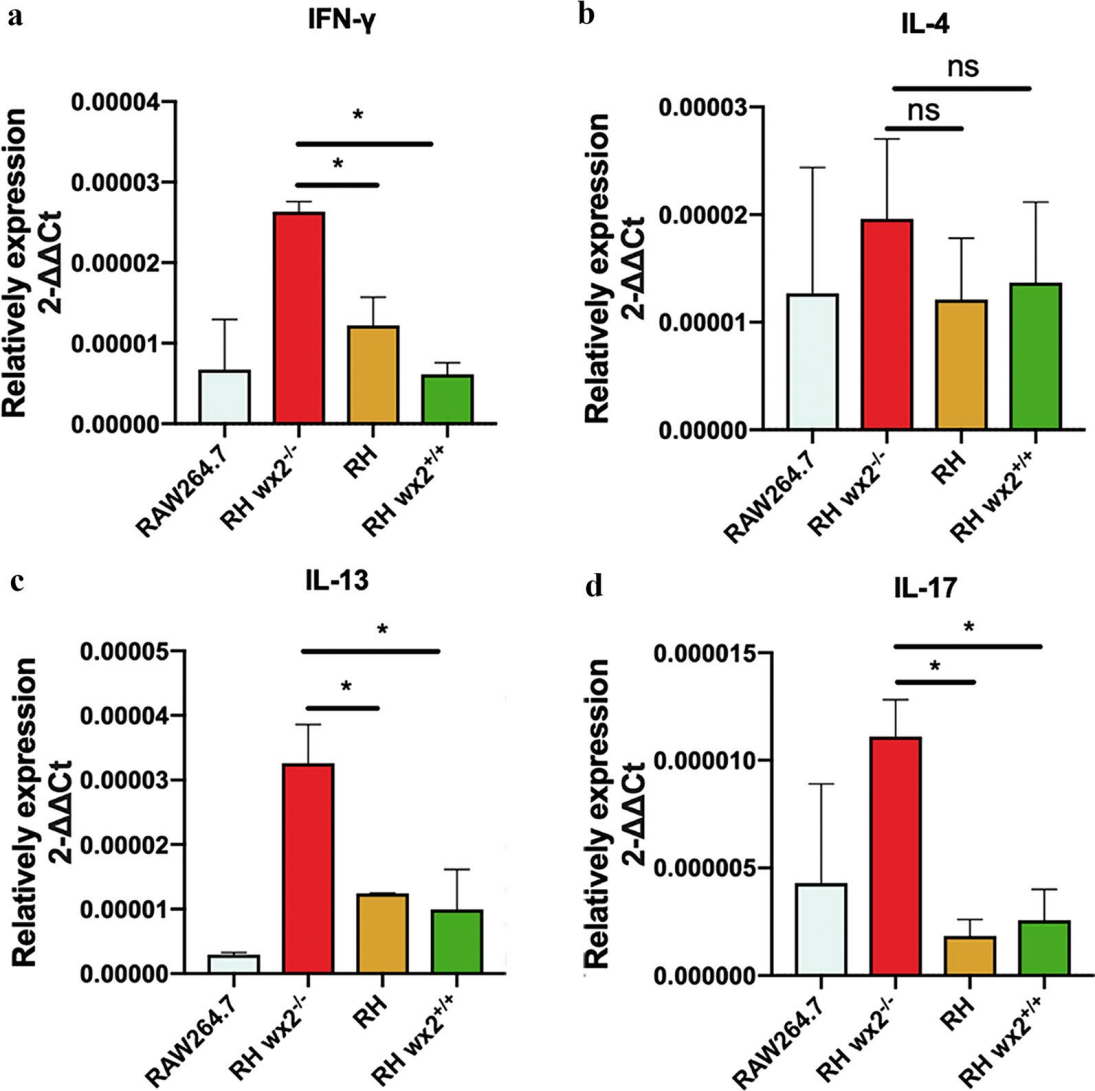
*wx2* gene influence host cell immune response in vitro. The mRNA expression of Th1-,Th2-, and Th17-related cytokines were measured by qPCR in infected RAW264.7 cells at 48 h post-infection. Total RNA was extracted, and RT-qPCR was performed with the indicated primer. mRNA expression of Th1-, Th2-, and Th17-related cytokines of IFN-γ (**a**), IL-4 (**b**), IL-13 (**c**), and IL-17(**d**) were detected

In infected RAW264.7 cells, the levels of IL-6 (data not shown) and IFN-γ in the cells infected with RH^*wx2−/−*^ were significantly higher than those of RH and RH^*wx2*+*/*+^ strains at 48 h post-infection (Fig. 5a). Interestingly and consistent with previous findings, the level of IL-4 and IL-13 cytokines were also elevated accordingly in RH^*wx2−/−*^ strains (Fig. 5b, c), In addition, the Th17-type cytokine IL-17 was also significantly increased in the RH^*wx2*-*/*-^ strains (Fig. 5d). Our data showed that *wx2* inhibited the immune response and the production of cytokines of the host.

## Discussion

When *T. gondii* invades the host cells, the host activates a specific immune response to fight against the parasite, and interferon gamma (IFN-γ) is the most important cytokine in *T. gondii* elimination. This cytokine is mainly produced by NK cells, macrophages, T cells, and neutrophils [34, 35]. As one of the most successful parasites, *T. gondii* can parasitize in more than 140 animal species, including humans [20]. After *T. gondii* invades the host, in order to ensure that it is not cleared by the host’s immune system, it needs to establish a balance with the host, so it has evolved a series of mechanisms to escape the host’s immunity.

Pyroptosis plays an important role during pathogenic infection. It not only has a pro-inflammatory effect, amplifying inflammation through chemotactic reactions, but can also eliminate pathogens by promoting the death of infected cells, and plays a protective role in the infected body. Kuriakose et al. found that the influenza virus induced pyroptosis of infected cells through the NLRP3 pathway and aggravated the inflammatory response in the lungs [36]. After the host is infected with hepatitis B virus (HBV), the virus blocks pyroptosis by inhibiting caspase-1 and avoiding being cleared by hepatic macrophages, thereby achieving immune tolerance [37]. Studies have found that dengue virus infection can induce pyroptosis of human monocytes by activating caspase-1, thus playing a role in pathogen elimination in the body [38]. Carvalho et al. confirmed that parasite infections including *Leishmania*, *Plasmodium*, and *T. gondii* can activate inflammasomes such as NLRP3, induce pyroptosis of infected cells, and remove pathogens [39]. It has been reported that both human and murine NLRP1 can specifically recognize a cytoplasmic serine protease inhibitor of *Bacillus anthracis*, *Shigella,* fungi, and *T. gondii* and activate caspase-1 to initiate the process of pyroptosis [40, 41]. NAIP2 can recognize the rod-shaped protein of *T. gondii* [42, 43], activating caspase-1 to initiate the classical pyroptosis pathway. Another study found that the P2X7R/NLRP3 pathway plays an important role in IL-1β secretion and inhibition of *T. gondii* proliferation in infected cells [44]. Under cellular pathophysiological conditions, ATP released from dying cells enhances the activation of P2X7R, thereby upregulating the NLRP3 inflammasome, which promotes the secretion of IL-1β, thereby controlling the proliferation of *T. gondii* [44], revealing that the NLRP3 inflammasome is involved in the activation mechanism of *T. gondii* infection and its protective effect on the body.

Based on our previous studies, we found that the *wx2* gene is associated with the virulence of *T. gondii* strains and may be involved in regulating the host immune response [32]. In this study, the virulence assay of mice revealed that the survival time for mice infected with *wx2* gene knockout strain RH^*wx2−/−*^ was significantly longer than that for the mice infected with the wild-type RH strain. The survival time for mice was significantly shortened after the *wx2* gene was restored, confirming that the *wx2* gene is a virulence-related gene. Studies have found that the production of IL-1β contributes to host control of *T. gondii* infection [45, 46], and our experiments showed that the mRNA and protein expression levels of IL-1β and IL-1β p17 in the infected RH^*wx2−/−*^ group were significantly higher than those of the RH wild strain and the RH^*wx2*+*/*+^ strain, and the activation levels of NLRP3, caspase-1, and GSDMD were significantly higher in the RH^*wx2−/−*^ group than those of the wild RH strain and the RH^*wx2*+*/*+^ strain group. Both in vitro and in vivo results showed that the expression levels of NLRP3 and caspase-1 p20 in the RAW264.7 cells and lymph nodes of mice infected with the RH^*wx2−/−*^ were higher than those in the infected RH strain and RH^*wx2*+*/*+^ strain. In addition, the phosphorylation level of NF-κB (p65) protein in the RH^*wx2−/−*^ group was higher than that in wild-type RH and RH^*wx2*+*/*+^ strains after infection for 48 h in vitro.

Therefore, IFN-γ is a critical factor in host protective immunity against *T. gondii* infection [47, 48], and Th1-related cytokines play a crucial role in the antimicrobial host immune mechanisms [49]. Consistent with previous experimental results, our research showed that *wx2* can avoid the host’s immune response by inhibiting the production of IFN-γ and IL-6, accomplishing immune evasion of the parasites to some extent. Interestingly, and consistent with the previous findings, Th2-type cytokines such as IL-4, IL-13, and Th17-type cytokine IL-17 were significantly higher in the RH^*wx2*+*/*+^ strain than in other groups. Our results suggested that *wx2* can achieve the immune evasion of *T. gondii* by inhibiting the host's immune response, thereby ensuring its invasion and reproduction in host cells. Our results confirmed that *wx2* gene knockout can promote the occurrence of host cell pyroptosis, thereby inhibiting the parasitism and dissemination of *T. gondii* in cells and accelerating the elimination by the host. It is suggested that the *wx2* gene may promote its proliferation and survival in the host by inhibiting the activation of the NLRP3 inflammasome, caspase-1, and GSDMD, and inhibiting the classical pyroptotic pathway. In addition, we found that the mRNA level of *wx2* expression in the complemented strain RH^*wx2*+*/*+^ could not be completely restored to the wild-type strain, which may be related to the CRISP/cas9 technology itself. When a gene is repeatedly knocked out and recovered, it will affect the expression ability of the gene itself, resulting in a difference from the wild type, but the corresponding phenotype can still be observed.

Since macrophages are one of the main cell types infected by *T. gondii *[50], therefore, macrophage pyroptosis may be a host mechanism to prevent parasite proliferation in the host. In addition, cytokines released by pyroptotic macrophages may attract other immune cells such as dendritic cells to fight infection. Therefore, *T. gondii*-induced pyroptosis of these cells can also inhibit the spread of *T. gondii*. Our next experiments also need to detect the level of pyroptosis of other immune cells to further reveal their anti-*Toxoplasma* mechanisms. But, in general, the *wx2* gene is a virulence-related gene of *T. gondii,* and it may participate in the immune regulation of the host by inhibiting the classical pathway of pyroptosis. In addition, since *wx2* was expressed not only in the RH strain but also in the ME49 strain, it suggests that *wx2* was expressed in both the acute and chronic phases, and can be used as a potential target for *T. gondii* detection.

## Conclusion

*wx2* is a virulence-related gene of *T. gondii,* and it may be regulate the host immune response by inhibiting the pyroptosis pathway.

## Supplementary Information

**Additional file 1: Figure S1.** Construction of the complementary strain. (a) Frame construction diagram of *wx2* complementing strain. Insertion of the *wx2* fragment into the Ptub::GOI::CAT plasmid and obtaining a vector contain tub promoter, CDS terminator of the *wx2* gene, and CDS region of the CAT gene. (b) Identification of the complementary strain of RH^*wx2+/+*^, by the detection of the insertion of *wx2* into the RH^*wx2-/-*^ strain. Lane1, RH^*wx2-/-*^ strain as a positive control. Lane 2-7, clone 1-6 from pTub::GOI::CAT - wx2 plasmid transformation to RH^*wx2-/-*^ strain. *wx2* fragment was detected in lane 5, namely, clone 4, which meant successful construction and screening of RH^*wx2+/+*^ strain. (c) Identification of the *wx2* expression at the mRNA level in the RH^*wx2-/-*^, RH, and RH^*wx2+/+*^ strains. The *wx2* expression level of the RH^*wx2+/+*^ strain was higher than that of the knockout RH^*wx2-/-*^ strain but lower than that of the wild-type RH.

## Data Availability

Data sharing is not applicable to this article as no datasets were generated or analyzed during the current study.

## Abbreviations

*T. gondii*: *Toxoplasma gondii*
Th: Helper T lymphocytes
IL: Interleukin
NK: Natural killer
IFN: Interferon
PVM: Parasitophorous vacuole membrane
ROP: Rhoptries
GRA: Granules
STAT: Signal transducer and activator of transcription
LPS: Lipopolysaccharide
TNF: Tumor necrosis factor
TLR: Toll-like receptor
TgIST: Toxoplasma inhibitor of STAT1-dependent transcription;
GSDMD: Gasdermin D
NLR: Nod-like receptor
PAMP: Pathogen-associated molecular patterns
DAMP: Damage-associated molecular patterns
HAMP: Hepcidin antimicrobial peptide
PCR: Polymerase chain reaction
SPF: Specific-pathogen-free
qRT-PCR: Quantitative real-time PCR
HBV: Hepatitis B virus
