# Supplementary figures and images for: Virulence-related gene wx2 of Toxoplasma gondii regulated host immune response via classic pyroptosis pathway
Source: Parasit Vectors. 2022 Dec 5;15:454. doi: 10.1186/s13071-022-05502-5 (PMC9724370; doi:10.1186/s13071-022-05502-5)

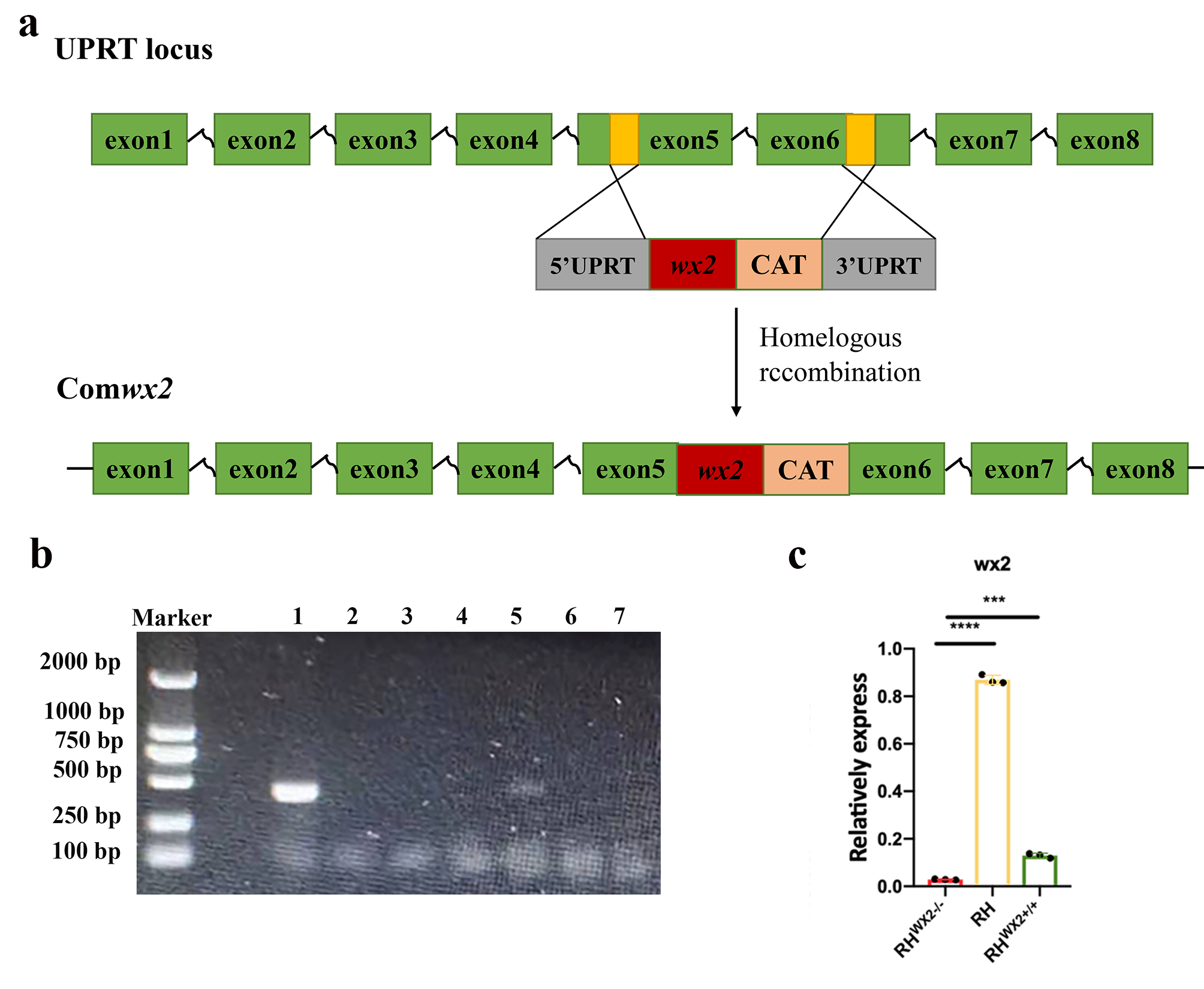

Supplement: Supplementary file 1 — Additional file 1: Figure S1. Construction of the complementary strain. (a) Frame construction diagram of wx2 complementing strain. Insertion of the wx2 fragment into the Ptub::GOI::CAT plasmid and obtaining a vector contain tub promoter, CDS terminator of the wx2 gene, and CDS region of the CAT gene. (b) Identification of the complementary strain of RHwx2+/+, by the detection of the insertion of wx2 into the RHwx2-/- strain. Lane1, RHwx2-/- strain as a positive control. Lane 2-7, clone 1-6 from pTub::GOI::CAT - wx2 plasmid transformation to RHwx2-/- strain. wx2 fragment was detected in lane 5, namely, clone 4, which meant successful construction and screening of RHwx2+/+ strain. (c) Identification of the wx2 expression at the mRNA level in the RHwx2-/-, RH, and RHwx2+/+ strains. The wx2 expression level of the RHwx2+/+ strain was higher than that of the knockout RHwx2-/- strain but lower than that of the wild-type RH. [file 13071_2022_5502_MOESM1_ESM.tif]
